# A phase 1 study of PF-07260437, a B7-H4 × CD3 bispecific T-cell engager, in patients with advanced or metastatic breast, ovarian, and endometrial cancer

**DOI:** 10.1007/s10637-026-01614-2

**Published:** 2026-05-22

**Authors:** Fengting Yan, Marcia Cruz-Correa, Jennifer Specht, Edward Wenge Wang, Jinyu Lu, Hatem Soliman, Amy Jackson-Fisher, Szu-Yu Tang, Sibo Jiang, Christophe Le Corre, Meng Li, David Sommerhalder

**Affiliations:** 1 Swedish Cancer Institute, 1221 Madison Street, Seattle, WA 98104 USA; 2 Pan American Center for Oncology Trials, 150 Av. Centro Médico, San Juan, PR 00935 USA; 3 Fred Hutchinson Cancer Center, 1100 Fairview Ave N, Seattle, WA 98109 USA; 4 City of Hope Comprehensive Cancer Center, 1500 E Duarte Rd, Duarte, CA 91010 USA; 5 Montefiore Einstein Comprehensive Cancer Center, 1695 Eastchester Rd, Bronx, NY 10461 USA; 6 Moffitt Cancer Center, 12902 USF Magnolia Drive, Tampa, FL 33612 USA; 7 Pfizer, Torrey Heights, 11202 El Camino Real, San Diego, CA 92130 USA; 8 Pfizer, 181 Oyster Point Blvd, South San Francisco, CA 94080 USA; 9 NEXT Oncology, 2829 Babcock Rd Suite 300, San Antonio, TX 78229 USA

**Keywords:** B7-H4, Bispecific, T-cell engager, First-in-human trial, Solid tumours

## Abstract

**Summary:**

B7-H4 is overexpressed in various cancers, making it a promising target for cancer immunotherapy. This phase 1, open-label, first-in-human study in patients with advanced/metastatic breast, ovarian and endometrial cancer evaluates the safety, pharmacokinetics (PK), and anti-tumour activity of PF-07260437, a novel B7-H4xCD3 bispecific T-cell engager. Enrolled patients received escalating doses of PF-07260437 (with/without priming dose) subcutaneously every two weeks, with a starting dose of 100 µg. Primary objectives included determination of maximum tolerated dose (MTD)/recommended dose for expansion (RDE), safety and tolerability. Secondary objectives included PK parameters and immunogenicity. Bayesian logistic regression model (BLRM) was used to guide dose escalation. Thirty patients with advanced/metastatic breast, ovarian and endometrial cancer received the study treatment during dose escalation; all were female (median age, 61.0 [41–75] years). Four patients (13.3%) experienced DLTs (alanine aminotransferase increased, aspartate aminotransferase increased, blood bilirubin increased, cytokine release syndrome). Overall, 29 (96.7%) patients experienced 202 treatment-related TEAEs (no grade 4/5 TEAEs); no TEAE resulted in study discontinuation. No objective response was observed. Disease control rate (DCR) was 33.3% (95% CI: 17.3%, 52.8%). PF-07260437 ≥ 300 μg resulted in exposures in the theoretic efficacious range, with a transient increase in serum cytokines levels following the first dose. The study was terminated by the Sponsor based on the overall assessment of the observed clinical safety, preliminary efficacy, pharmacokinetic and pharmacodynamic data. The MTD was not reached. PF-07260437 was tolerable in patients with breast, ovarian, and endometrial cancers with manageable TEAEs.

**Trial Registration Number:**

NCT05067972.

**Registration date:**

5 October, 2021

**Supplementary Information:**

The online version contains supplementary material available at 10.1007/s10637-026-01614-2.

## Highlights

- This study evaluated the safety, PK, immunogenicity, and anti-tumour activity of PF-07260437 in patients with solid tumours
- Overall, 6 treatment-related DLTs were reported in 4 patients, which eventually resolved
- DCR across cohorts was 33.3% (10/30)
- PF-07260437 was tolerable in patients with breast, ovarian, and endometrial cancers with a manageable safety profile

**Supplementary Information:**

The online version contains supplementary material available at 10.1007/s10637-026-01614-2.

## Introduction

CD3 bispecific T-cell engagers are designed to simultaneously bind to specific tumour-expressed antigens on cancer cells and T cell activation receptor CD3 [1, 2], leading to MHC-independent T cell activation without the requirement of costimulatory signals [2, 3]. As a result of this activation, T cells release perforin and granzyme B which leads to killing of cancer cells [3]. In the last decade, research on CD3 bispecific antibody therapies targeting B cell-specific or plasma cell-specific antigens such as CD19, CD20, B-Cell Maturation Antigen (BCMA), or G-protein coupled receptor, Class C, Group 5, Member D (GPRC5D) has demonstrated considerable efficacy in treatment of B cell malignancies [1]. While blinatumomab was the first CD3 bispecific antibody to be approved for the treatment of refractory/relapse B-lineage acute lymphoblastic leukaemia (ALL) in 2014 and later for specific patient groups in 2018 and 2024 [4–6], the recent success of this treatment strategy is demonstrated by the approval of therapeutics such as mosunetuzumab (anti-CD20 x CD3), epcoritamab (anti-CD20 x CD3), teclistamab (anti-BCMA x CD3), talquetamab (anti-GPRC5D x CD3), and glofitamab (CD-20 × CD3) in relapsed/refractory haematological malignancies [7–12]. Based on their favourable safety and efficacy profile in relapse/refractory settings, the role of these antibodies is now being investigated for first-line treatment of multiple myeloma and other haem malignancies [1].

On the other hand, evidence of success of CD3 bispecific antibodies in patients with solid tumours is only beginning to emerge. In 2021, a phase 1 clinical trial evaluating pasotuxizumab, a bispecific antibody targeting prostate-specific membrane antigen (PSMA) and CD3, in metastatic castration-resistant prostate cancer reported early evidence of efficacy, with > 50% reduction in prostate-specific antigen levels in 30% of patients enrolled [13]. Similarly, findings from a phase 2 clinical trial evaluating the safety and efficacy of tarlatamab, a CD3-targeting bispecific antibody against the Notch ligand DLL3, revealed an encouraging objective response rate (ORR) of 40% (97.5% confidence interval [CI]: 29, 52) in patients receiving a 10 mg dose, and 32% (97.5% CI: 21, 44) in those receiving a 100 mg dose along with a manageable safety profile in patients with previously-treated small cell lung cancer [14]. Based on this, tarlatamab has received accelerated FDA approval in 2024 for use in patients with extensive stage SCLC [15].

B7-H4 is an immune checkpoint molecule that negatively regulates immune responses and is overexpressed in various cancers, including gynaecological cancers, making it a promising target for cancer immunotherapy [16]. Several genetic as well as immunohistochemistry studies evaluating B7-H4 levels in patient tissue samples have revealed considerable overexpression of B7-H4 in ovarian and breast cancer cells, along with minimal to no expression in healthy or benign tissues [17–21]. B7-H4 overexpression has also been reported in 48% of patients with stage 1 ovarian cancer, suggesting the potential effectiveness of B7-H4 based therapy in early-stage disease [22]. Furthermore, an increased proportion of B7-H4 staining in malignant endometrial tissues versus normal epithelium has also been observed [23]. To date, preclinical studies utilising humanized mouse models have provided preliminary evidence of the anti-tumour activity of B7-H4 based bispecific antibodies, with one study reporting 70% reduction in tumour size after administration of a B7-H4 x CD3 bispecific antibody and another demonstrating 79% inhibition in tumour growth after treatment with CLN-418, a B7-H4 × 4-1BB antibody [24, 25]. These outcomes support the rationale behind several ongoing or recently completed first-in-human (FIH) clinical trials evaluating the efficacy and safety of B7-H4 based bispecific antibodies in patients with various types of solid tumours. The findings of most of these clinical trials are yet to be published (NCT05180474, NCT06126666, NCT05306444).

PF-07260437 is a novel B7-H4 x CD3 bispecific T-cell engager that aims to address this significant unmet medical need in cancer treatment by directly regulating tumour-localized T cell cytotoxic responses. In this phase 1, open-label, multi-centre, FIH study (ClinicalTrials.gov, NCT05067972), the safety, pharmacokinetics, immunogenicity, and anti-tumour activity of PF-07260437 was evaluated in patients with advanced or metastatic breast, ovarian and endometrial cancer. Preclinically, PF-07260437 demonstrated B7-H4 expression-dependent antitumour activity against a panel of breast cancer cell line xenografts and patient-derived xenografts grown in immune-compromised mice that were engrafted with human T cells [26]. To the best of our knowledge, this is the first report of findings from a clinical trial investigating the role of a B7-H4 x CD3 bispecific T-cell engager in solid tumours.

## Methods

### Study design and population

The original study design consisted of dose escalation (Part 1) and dose expansion (Part 2) cohorts. However, the study was terminated by the Sponsor on July 27, 2023 based on the overall assessment of the observed clinical safety, preliminary efficacy, pharmacokinetic and pharmacodynamic data. Enrolment into Part 1 was discontinued and Part 2 was not initiated. Notably, this decision was not due to major safety concerns or requests from any regulatory authorities.

Patients ≥ 18 years of age with a histological or cytological diagnosis of locally advanced or metastatic advanced breast, ovarian, or endometrial cancer that is resistant to standard therapy or for which no standard therapy is available; Eastern Cooperative Oncology Group Performance Status (ECOG PS) 0 or 1; with adequate liver, renal, and bone marrow function, were included in this study. Patients with a high risk of short-term life-threatening complications, a history of active malignancy within 3 years prior to enrolment, or clinically significant cardiovascular disease risk within 6 months prior to enrolment were excluded. Pre-selection for B7-H4 biomarker levels was not conducted.

### Schedule and dosing

**Fig. 1:**
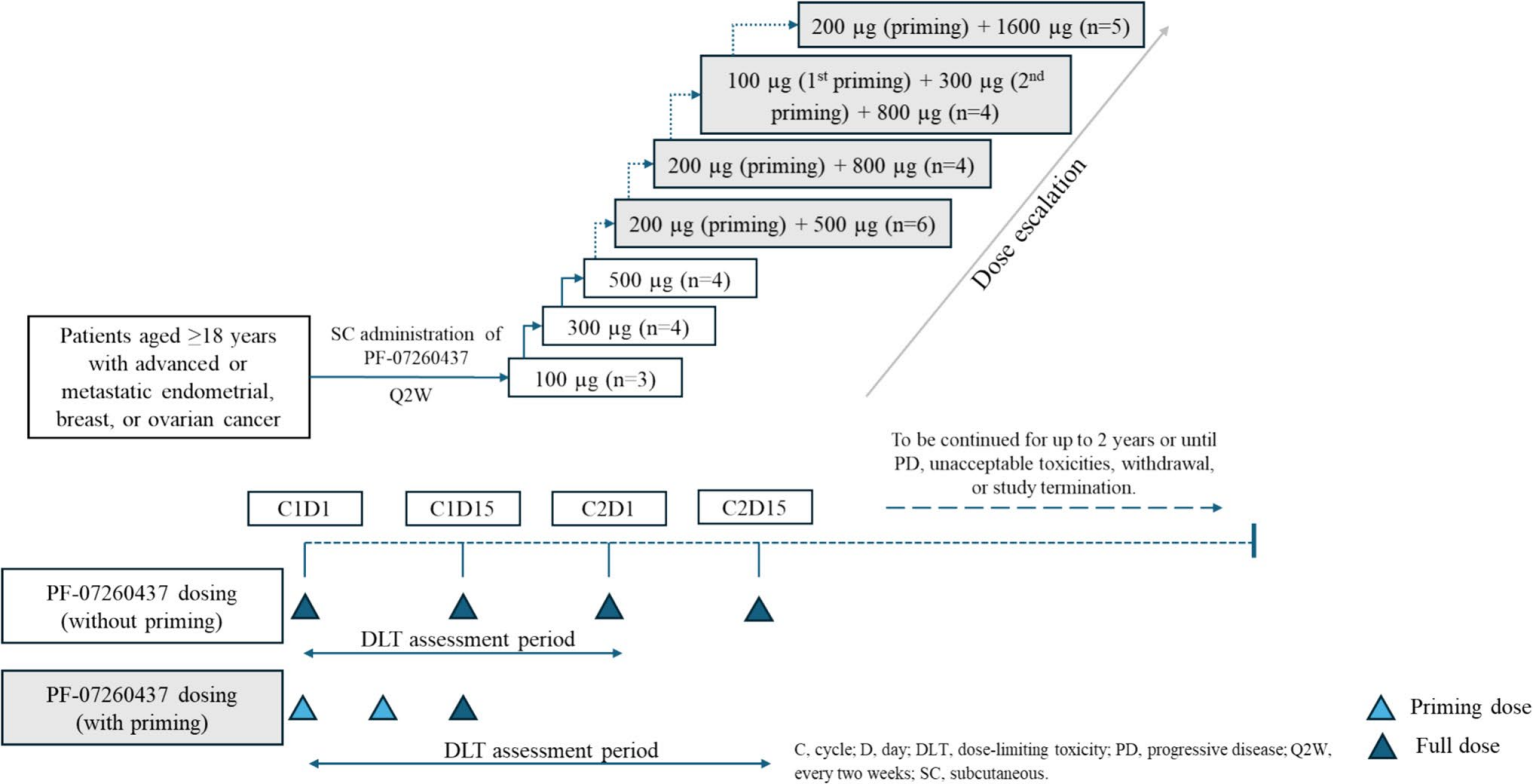
Overview of study design and dosing schedule

Escalating doses of PF-07260437 were administered subcutaneously (SC) every two weeks (Q2W), starting with an initial dose of 100 µg. While the maximum allowable dose increment was 200% (threefold increase), it could be reduced to 100% (twofold increase) following the observation of a DLT or two ≥ Grade 2 clinically significant treatment-related treatment-emergent adverse events (TEAEs). Priming strategy was implemented to mitigate the potential cytokine release syndrome (CRS). Furthermore, if a dose induced ≥ Grade 2 CRS (lasting for > 24 h) in 1 or more patient despite treatment with tocilizumab and/or vasopressors, a priming dose was to be administered to sensitize the immune system and reduce the rate and intensity of CRS. A single priming dose was to be administered on Day 1 of the cycle at 1 dose level below which the ≥ Grade 2 CRS event was observed, followed by full dose (100% higher) on day 15; a two step-up priming doses approach was investigated, with the 2 priming doses administered on Day 1 and 8 of the cycle. Patients continued to receive PF-07260437 for up to 2 years or until disease progression (defined by immune related response criteria derived from response evaluation criteria in solid tumours [irRECIST] v1.1), unacceptable toxicities, a decision by the patient or investigator to discontinue treatment, or study termination. A Bayesian logistic regression model (BLRM) was used to guide dose escalation. An overview of the study design and dosing schedule is provided in Fig. 1.

### Study objectives and endpoints

The primary objective of the study was to determine the maximum tolerated dose (MTD) and the recommended dose for expansion (RDE) of PF-07260437, as well as to assess the safety at increasing dose levels. Safety was evaluated in terms of incidence of dose-limiting toxicities (DLTs), and TEAEs were characterized by type, frequency, severity (as graded by National Cancer Institute [NCI] Common Terminology Criteria for Adverse Events [CTCAE] v5 or American Society for Transplantation and Cellular Therapy [ASTCT] CRS grading system) [27] timing, seriousness, and relationship to therapy. For drug administration without a priming strategy, DLTs were assessed during the first 28 days. For drug administration with priming, the DLT assessment period was extended to account for the priming doses, thereby extending the assessment period by 14 days. A patient was considered evaluable for DLT if they either received all the planned doses of PF-07260437 and all scheduled safety assessments during the DLT observation period or experienced a DLT.

The secondary objectives of the study included the assessment of pharmacokinetic (PK) parameters and immunogenicity, measured by incidence and titres of anti-drug antibody (ADA) and neutralizing antibody (NAb) against PF-07260437. Exploratory objectives included the evaluation of preliminary antitumour activity based on investigator-assessed ORR (complete response [CR] + partial response [PR]) the duration of response (DOR) as per RECIST v1.1 and irRECIST, the identification of biomarkers that may predict response to therapy, and the evaluation of cytokines that may demonstrate pharmacodynamic modulation in serum samples.

### Statistical analysis

No formal hypothesis testing was planned for this study. Dose escalation was guided by a Bayesian analysis of DLT for PF-07260437 with the escalation with overdose control (EWOC) principle. A two-parameter logistic regression model was used to evaluate the probability of a patient experiencing a DLT at a given dose. A dose was only used for newly enrolled patients if the risk of overdosing at that dose was less than 25%. After each cohort, the posterior distribution for the risk of DLT was estimated for each dose of interest and the recommendations from the Bayesian logistic regression model (BLRM), along with safety, laboratory, PK, biomarker, and other endpoints were used to guide dose escalation and MTD/RDE determination. For continuous endpoints, median (range) was estimated; percentages for binary endpoints were provided along with the corresponding 2-sided 95% confidence intervals (CIs) using exact method.

### Declaration of generative AI and AI-assisted technologies in the writing process

The methods section of the article was drafted using Pfizer’s AI-based tool (Medical AI Assistant [MAIA]). After using this tool/service, the content was reviewed and edited (as needed), and the authors take full responsibility for the content of the publication.

## Results

### Patients disposition and baseline characteristics

Out of 38 patients who were screened for enrolment, 30 were enrolled and assigned to treatment across 8 centres in the USA and Puerto Rico (Online Resource:Supplementary Fig. S1**)**. Patients received escalating doses of PF-07260437 as per dosing schedule provided in Fig. 1. All patients eventually discontinued the treatment, with the majority discontinuing due to progressive disease (56.7%). All patients enrolled in this study were female, with a median age of 61.0 years (range: 41–75 years). The majority of the patients were White (83.3%), not of Hispanic or Latino ethnicity (70.0%), and had a baseline ECOG PS of 0 (66.7%) (Online Resource:Supplementary Table S1**)**. Most of the patients (46.7%) had a primary diagnosis of ovarian cancer; 23.3% had hormone receptor (HR) + human epidermal growth factor receptor 2 negative (HER2)- breast cancer, 16.7% had endometrial cancer, 10.0% had triple negative breast cancer (TNBC), and 3.3% had HER2 + breast cancer (Online Resource:Supplementary Table S1**)**. The median duration of exposure to PF-07260437 was 6.7 weeks (range: 0.1–35.1 weeks), with the majority (86.7%) being exposed for ≤ 12 weeks.

### Safety

Out of 30 patients assigned to treatment, 20 (66.7%) were considered evaluable for DLTs. In total DLTs were reported in four patients (13.3%) (Online Resource: Supplementary Table S2), all eventually resolved. One patient treated at 500 µg Q2W of PF-07260437 experienced Grade 3 CRS. Another patient treated at 200 µg (priming) + 1600 µg Q2W of PF-07260437 experienced Grade 3 alanine transaminase (ALT) increase. Two patients treated at 100 µg (priming) + 300 µg (second priming) + 800 µg Q2W of PF-07260437 experienced DLTs: One patient with Grade 3 aspartate transaminase (AST) increase; another patient with Grade 2 increase in serum bilirubin levels, Grade 1 AST increase, and 2 episodes of ALT increase (Grade 2 and Grade 3).

Overall, 346 all-causality TEAEs were reported in 30 (100.0%) patients across all dose levels, with 202 TEAEs in 29 (96.7%) patients being treatment-related. Among all-causality TEAEs reported in this study, Grade 3 and 4 TEAEs were observed in 19 (63.3%) patients and 1 (3.3%) patient, respectively (Online Resource: Supplementary Table S3). Grade 3 treatment-related TEAEs were reported in 21 (70.0%) patients, with the most frequent being increases in ALT (46.7%), AST (30.0%), and alkaline phosphatase (23.3%) (Online Resource: Supplementary Table S4). Furthermore, 2 (6.7%) patients in PF-07260437 200 µg (priming) + 500 µg cohort experienced Grade 5 TEAEs of dyspnoea (Online Resource: Supplementary Table S3) and acute hepatic failure. No Grade 4/5 treatment-related TEAEs were reported (Online Resource: Supplementary Table S4) and no patient discontinued the study due to TEAEs. Treatment-related TEAE stratified by Grade (Grade 1–2 and ≥ Grade 3) and dose level included in Table 1.

**Table 1.**
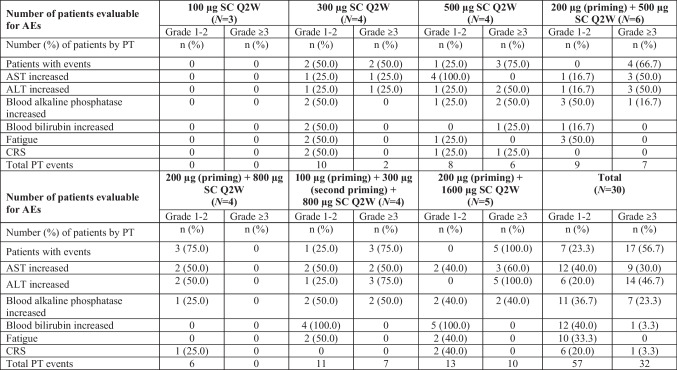
Treatment-related TEAEs (Grade 1–2 and Grade ≥ 3) with specific PTs by PT and maximum CTCAE grade (all cycles, safety analysis set)

Treatment related means related to any study intervention. Patients reporting more than 1 treatment-related TEAE within a PT are counted only once in that PT at the maximum grade. Treatment-related TEAEs are sorted in descending frequency by total column of treatment group. Includes data up to earliest of 30 days post last dosing date and 1 day prior to new anti-cancer therapy. MedDRA v26.1 coding dictionary applied and severity graded by NCI CTCAE version 5

*AE* Adverse event, *ALT* Alanine aminotransferase, *AST* Aspartate aminotransferase, *CRS* Cytokine release syndrome, *CTCAE* Common Terminology Criteria for Adverse Events, *DLT* Dose-limiting toxicity, *MedDRA* Medical Dictionary for Regulatory Activities, *NCI* National Cancer Institute, *PT* Preferred Term, *Q2W* every 2 weeks, *SC* subcutaneous, *TEAE* treatment emergent adverse event

Interestingly, no grade 3 CRS was reported in dose escalation cohorts with priming strategy. On the other hand, transient liver enzyme increases were observed in dose escalation cohorts with or without priming.

### Anti-tumour activity

Confirmed CR/PR or immune-related CR/PR were not observed in any patient. (Table 2). The disease control rate (DCR) across cohorts was 33.3% (95% CI: 17.3%, 52.8%), with 8 (26.7%) patients achieving stable disease.

**Table 2:** Summary of best overall response and objective response (confirmed) based on investigator assessment (RECIST v1.1) in all patients (full analysis set)

Response	Overall (*n* = 30)	100 µg (*n* = 3)	300 µg (*n* = 4)	500 µg (*n* = 4)	200 µg (priming) + 500 µg (*n* = 6)	200 µg (priming) + 800 µg (*n* = 4)	100 µg (1st priming) + 300 µg (2nd priming) + 800 µg (*n* = 4)	200 µg (priming) + 1600 µg (*n* = 5)
CR	0 (0)	0 (0)	0 (0)	0 (0)	0 (0)	0 (0)	0 (0)	0 (0)
PR	0 (0)	0 (0)	0 (0)	0 (0)	0 (0)	0 (0)	0 (0)	0 (0)
SD	8 (26.7)	0 (0)	2 (50.0)	1 (25.0)	2 (33.3)	0 (0)	2 (50.0)	1 (20.0)
Non-CR/Non-PD	2 (6.7)	1 (33.3)	0 (0)	0 (0)	0 (0)	0 (0)	1 (25.0)	0 (0)
PD	16 (53.3)	2 (66.7)	2 (50.0)	3 (75.0)	3 (50.0)	3 (75.0)	0 (0)	3 (60.0)
NE	4 (13.3)	0 (0)	0 (0)	0 (0)	1 (16.7)	1 (25.0)	1 (25.0)	1 (20.0)
ORR, % (95% CI)	0.0 (0.0, 11.6)	0.0 (0.0, 70.8)	0.0 (0.0, 60.2)	0.0 (0.0, 60.2)	0.0 (0.0, 45.9)	0.0 (0.0, 60.2)	0.0 (0.0, 60.2)	0.0 (0.0, 52.2)
DCR, % (95% CI)	33.3 (17.3, 52.8)	33.3 (0.8, 90.6)	50.0 (6.8, 93.2)	25.0 (0.6, 80.6)	33.3 (4.3. 77.7)	0.0 (0.0, 60.2)	75.0 (19.4, 99.4)	20.0 (0.5, 71.6)

All values are presented as n (%), unless stated otherwise

*CI* Confidence interval, *CR* Complete response, *DCR* Disease control rate, *NE* Not evaluated, *ORR* Objective response rate, *PD* Progressive disease, *PR* Partial response, *SD* Stable disease

### Pharmacokinetics and Immunogenicity

**Fig. 2:**
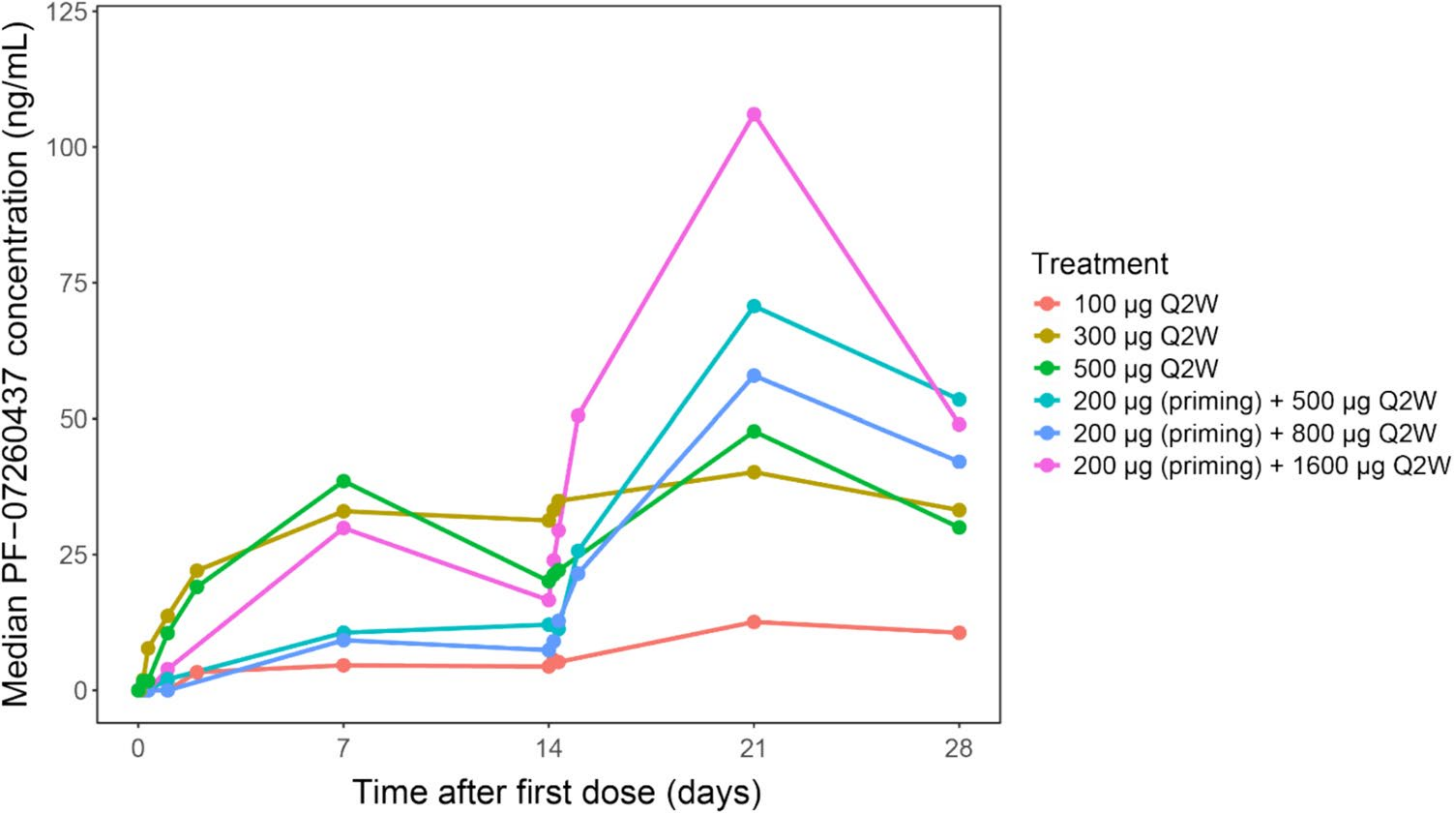
Median serum concentration–time profile of PF-07260437 from patients enrolled in the study. Q2W, every two weeks. The cohort of 100/300/800 µg was not presented due to different dosing schedule and missing PK sampling on day 14 and 21

In this study, an increase in exposure of PF-07260437 was observed with an increase in dose from 100 μg to 1600 μg over the first cycle. PF-07260437 showed prolonged absorption (T_max_ ~ 7 days) following SC injection, and the exposure was generally higher after the second dose, indicating expected accumulation. The steady state could not be determined due to patient dropout in later cycles. All the doses of ≥ 300 μg Q2W resulted in exposures achieving the theoretic efficacious range (≥ 12 ng/mL) as predicted by the preclinical model. The median PF-07260437 serum concentration time profiles from 24 patients with full PK profile over the first cycle are presented in Fig. 2. Out of the 26 ADA evaluable patients who had at least one post-treatment ADA sample, the incidence of ADA was 7 (26.9%) patients, and all of them were treatment-induced. Only one patient had positive baseline ADA against PF-07260437 but was not boosted following treatment.

### Cytokine analysis

**Fig. 3:**
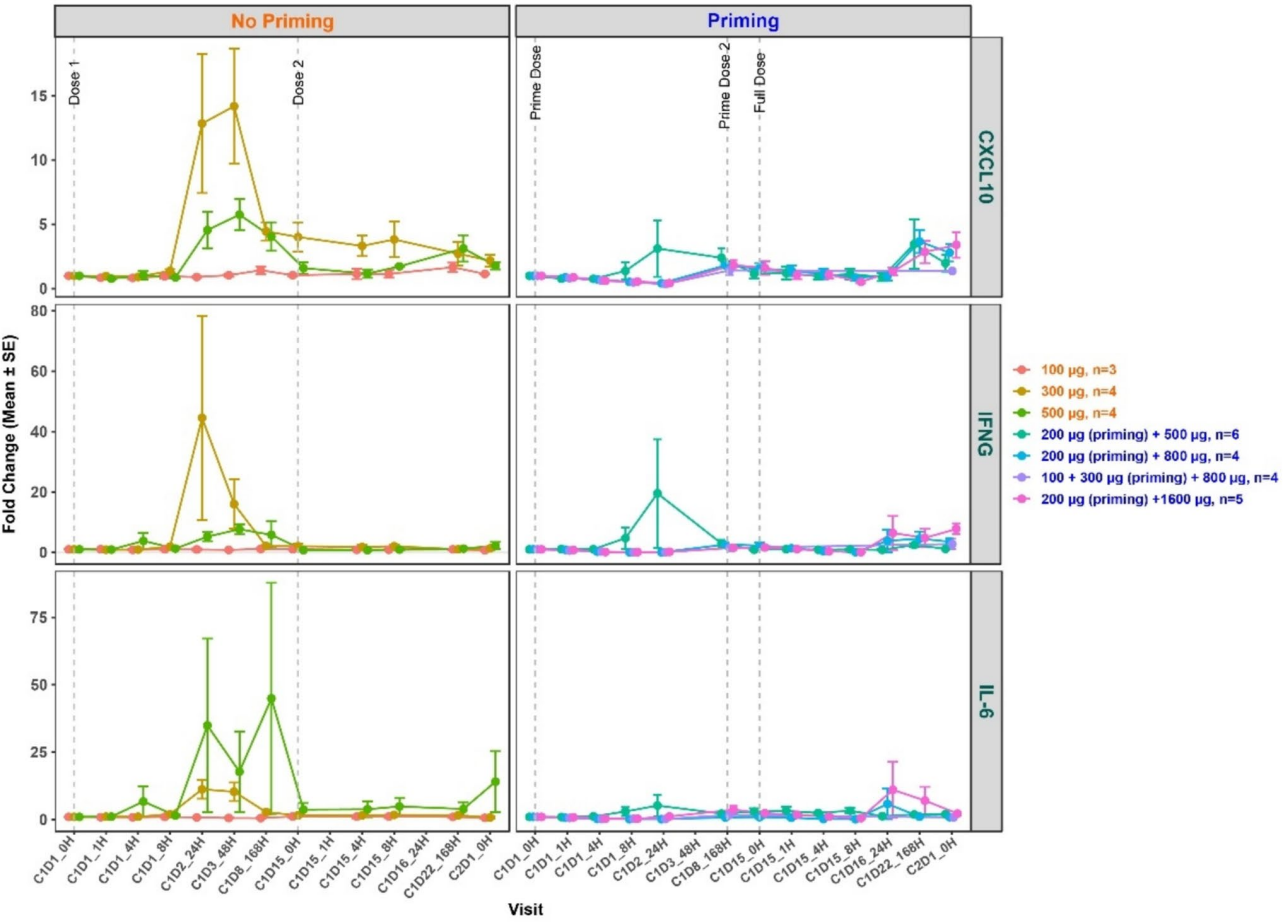
Mean fold change of serum cytokines in patients with or without priming doses (full analysis set). C, cycle; CXCL, chemokine ligand; D, day; H, hour; IFNG, interferon-gamma; IL, interleukin

Serum cytokines, including those produced by activated T cells, exhibited a transient increase following the first dose of PF-07260437 in patients who did not receive a priming dose **(**Fig. 3**)**. Elevated levels of chemokine ligand 10 (CXCL10), interferon-gamma (IFNG), and interleukin (IL)−6 were observed in the 300 µg and 500 µg monotherapy cohorts. Across these cohorts, mean cytokine levels increased over baseline by 14-fold in CXCL10 at C1D3 in the 300 µg cohort, 45-fold in IFNG at C1D2 in 300 µg cohort, and 45-fold in IL-6 at C1D8 in the 500 µg cohort. In patients who received priming dose(s), the increase in serum cytokine levels was lower, with a maximum mean fold increase over baseline of fourfold for CXCL10 at C1D22 in the 200 µg priming + 800 µg dose cohort, 20-fold in IFNG at C1D2 in the 200 µg priming + 500 µg dose cohort, and 11-fold in IL-6 in at C1D16 in the 200 µg priming + 1600 µg dose cohort. These findings supported that priming strategy may mitigate CRS risk of PF-07260437.

## Discussion

In this study, PF-07260437 was tolerable in patients with breast, ovarian, and endometrial cancers with manageable TEAEs and no patient discontinued treatment due to TEAEs. The study was terminated by the Sponsor based on the overall assessment of the clinical safety, efficacy, PK and pharmacodynamic data. The MTD was not reached. Overall, four patients experienced 6 treatment-related DLTs, which eventually resolved. The majority of DLTs occurred in cohorts receiving ≥ 1 priming dose, which included increase in bilirubin (Grade 2), AST (Grade 1 and 3), and ALT (Grade 2 and 3). From a safety perspective, the most expected TEAEs based on the mechanism of action (MOA) of CD3-targeting therapy was CRS. One Grade 3 CRS was observed in Cohort 3 at 500 µg SC Q2W, which was subsequently successfully mitigated by priming strategy. No additional CRS event ≥ Grade 2 was observed with either one-step or two-step priming approach in cohorts 4, 5, 6, and 7. No ICANS (immune effector cell-associated neurotoxicity syndrome) was reported. The most unexpected and frequent TEAEs was transient liver enzyme elevation, which was typically associated with the first priming dose or full dose and resolved within 2–3 weeks. Furthermore, re-exposure to the same dose level did not trigger the same AE. This transient liver enzyme elevation could not be mitigated by priming strategy. We hypothesize that transient transaminitis after the first priming dose or first full dose might be associated with initial transient cytokine release based on the mechanism of action and temporal correlation observed so far. While low B7-H4 expression in biliary ducts may exacerbate local inflammation in the liver, the exact mechanism leading to transaminitis remains unclear. In the present study, transaminitis/liver toxicity was frequent with PF‑07260437, with AST elevations in 40% of patients (grade ≥ 3, 30%) and ALT elevations in 20% (grade ≥ 3, 46.7%). Liver enzyme elevations were unpredictable and not dose dependent. For comparison, liver enzyme elevations have also been reported with other CD3‑bispecific therapies. In the MajesTEC-1 phase 1–2 study of teclistamab, increases in blood alkaline phosphatase were reported in 10.9% of patients with relapsed or refractory multiple myeloma [28]. Teclistamab may lead to hepatotoxicity, including fatalities. In clinical trials at the recommended dose, elevated AST occurred in 47% of patients (2.9% grade 3/4), and elevated ALT in 48% (3.8% grade 3/4) [29]. Similarly, in the TOWER phase 3 trial of blinatumomab in patients with acute lymphoblastic leukemia, grade ≥ 3 liver enzyme elevations were reported in 12.7% of patients [30]. In a phase 2 study of blinatumomab in patients with relapsed or refractory B‑precursor acute lymphoblastic leukemia, ALT increases were observed in 13% of patients, including grade 1–2 elevations in 6% and grade 3 elevations in 6% [31]. These data further indicate that liver toxicity might be associated with CD3-bispecific therapies. However, it remains unclear why the liver enzyme elevation observed with PF-07260437 was transient and why rechallenge and re-exposure at the same dose level did not result in a similar adverse event (liver enzyme elevation).

In general, PF-07260437 exposure increased in a dose-proportional manner, with the highest median peak concentrations observed in the 500 µg cohort. However, steady-state concentrations were not achieved in most patients due to dosing delays and discontinuation related to AEs or disease progression. Furthermore, PK parameters and NAb incidence and titre could not be evaluated, as the study was terminated and the dose expansion part of the study was not initiated. Peripheral cytokine analysis demonstrated early sign of peripheral inflammatory response with cytokine elevation. Further, it was observed that the priming could reduce the initial CRS spike. However, these observations cannot be directly translated to determine T-cell activation and antitumour activity. Clinical dose selection was informed by a translational pharmacodynamic (PD) model derived from mouse cell line xenograft (CLX) studies, assuming identical efficacious trimer concentrations (C_eff_) between mouse and human. This model incorporated assumptions of identical efficacious trimer levels, higher human tumour T‑cell density compared to mouse tumours, and comparable target antigen (TAA) density, and drug affinities to TAA and CD3. Based on these assumptions, the predicted human C_eff_ was 12 ~ 120 ng/ml (0.08–0.8 nM) at steady state. PF-07260437 administered at doses as low as 1.5–20 µg/kg QW were sufficient to achieve tumour stasis in mouse CLX studies [26]. In our study, patients were dosed at 1.5–20 µg/kg Q2W. Although the human dosing density is lower due to longer dosing intervals, the human exposure is expected to result in comparable efficacious trimer levels between human and mouse. However, the exposure was neither able to reach steady-state due to patient drop-out nor able to continue to escalate as limited by the DLT. As a result, the exposure reached the estimated low bound (≥ 12 ng/mL) in the initial cycles but not the high bound (≥ 120 ng/mL) of C_eff_.

Patients with breast, ovarian, and endometrial cancer were included in this study due to established differential overexpression of B7-H4 in malignant versus normal tissues across in these cancer types [17–23]. This observation, taken together with the preclinical evidence suggesting the potential of B7-H4 based therapeutics in cancer treatment, provided a strong rationale for evaluation of the role of PF-07260437 in this population. While several in vitro and animal studies have demonstrated that blocking of endogenous B7-H4 via blocking antibodies leads to restoration of anti-tumour T cell responses and delay in tumour growth, clinical data supporting the efficacy and safety of B7-H4 based therapies is limited [32–35].

Currently, several types of B7-H4-based therapeutics are still in development, including B7-H4 blocking antibodies, B7-H4 based bispecific antibodies, and B7-H4 based antibody–drug conjugates (ADCs). In a phase 1/2 study, *NC762*, a humanized IgG1κ monoclonal antibody targeting B7-H4, was reported to be safe and well-tolerated in patients with advanced or metastatic solid tumours; however, the study has been discontinued by the Sponsor due to limited efficacy, prioritizing the development of B7-H4 ADC (NCT04875806) [36]. Interim results from a phase 1 study evaluating the safety and efficacy of *FPA150*, a fully human antibody against B7-H4, have revealed a favourable safety profile (NCT03514121) in patients with advanced solid tumours [37]. Building upon the success of B7-H4 blocking antibodies, clinical trials evaluating B7-H4 based bispecific antibodies across various cancer types have also been initiated; however, most findings are yet to be published.

In our phase 1 study, the best overall response was SD (8 patients [26.7%]) with no patient achieving CR or PR, suggesting a lack of a strong antitumour activity (tumour shrinkage). This outcome is contrary to the preclinical finding, where more robust antitumour activity (tumour growth inhibition and regression) was observed [26]. For immunotherapy, clinical translatability of preclinical models is limited. Few animal models fully capture the complex nature of human TME. Although xenograft models combined with adoptive T-cell transfer (ACT) provide a quick platform to evaluate immunotherapy and CD3 engagers, they face significant limitations, including an immunodeficient environment (lacking mature T, B or NK cells), xenogeneic graft-versus-host disease (GvHD), and the absence of a proper tumour immune microenvironment, as well as T-cell exhaustion/dysfunction, poor persistence, trafficking failures. These limitations restrict the preclinical model's predictive value. Humanized mouse models (immunodeficient mice engrafted with human immune cells such as (PBMCs or, HSCs) are crucial for testing immunotherapies, offering a functional human immune system that mimics tumour-immune interactions. They enable testing of CD3 engagers and checkpoint inhibitors but also are limited by GvHD, high costs, and incomplete human cell maturation. Based on current analysis and staining, B7-H4 seemed differentiated and highly expressed on tumour cells rather than in the TME. These factors vary between patients and may not be reflected in preclinical models.

Furthermore, while CD3 bispecific antibodies have demonstrated robust clinical response in haematological malignancies such as lymphomas, leukaemia and multiple myeloma, the antitumour activity of CD3 bispecific engagers in solid tumours is limited [38]. Despite the broad expression of B7-H4 across multiple malignancies and its inverse association with patient prognosis and intratumoural T-cell infiltration, clinical translation of a B7-H4‑targeted bispecific T-cell engager in solid tumours remains challenging [39]. Bispecific T-cell engager therapies in solid tumours are hindered by on-target, off-tumour toxicities arising from antigen expression in healthy tissue. Toxicities such as B-cell or myeloid-cell depletion can lead to organ failure and death in solid tumours while in hematological cancers, such toxicities are reversible as long as hematopoietic stem cells are not targeted [40]. Furthermore, solid tumours often have relatively poor blood flow with sparse infiltration of T cells of impaired quality thereby preventing CD3 bispecific engagers from cross-linking T cells to tumour cells resulting in limited treatment efficacy. The immunosuppressive TME, enriched with inhibitory cell populations, further compromises effector T-cell quality and activity [41]. Collectively, these factors may help explain the lack of clinical efficacy observed with B7‑H4‑targeted bispecific T‑cell engager therapy in solid tumours.

The limitations of our PF-07260437 (B7-H4 × CD3 bispecific T-cell engager) phase 1 study include the phase 1 early dose-escalation, with small sample sizes across dose levels and cohorts. Interpretation of the trial results is limited given small sample size and single arm design, as well as transient, unexpected elevations in liver enzymes without a clear mechanism. The MTD was not reached, and limited antitumour activity was observed at the current dose levels. Though serum cytokines serve as surrogate and sensitive peripheral PD biomarkers, the study lacked convincing and specific tumour PD biomarker to connect peripheral systemic inflammatory response to direct antitumour activities in TME. In addition, two phase 1 studies evaluating the safety and efficacy of *GEN1047*, a B7-H4 x CD3 directing bispecific antibody, and *ABL103*, a B7-H4 × 4-1BB directing bispecific antibody, in solid tumours, including gynaecological tumours, are ongoing (NCT05180474, NCT06126666). Of note, another phase 1/2 study evaluating the safety and efficacy of *CLN-418*, a B7-H4 × 4-1BB bispecific antibody with strong preclinical evidence of efficacy in syngeneic tumour model in 4–1BB-humanized mice, has been completed, with results pending publication (NCT05306444) [25].

Nevertheless, to the best of our knowledge, this trial represents the first clinical results for a B7-H4 bispecific T-cell engager in solid tumours. These observations highlight key clinical development challenges of CD3 engager in solid tumours and provide a baseline and foundation for next generation molecule optimization and improvement. As results from other clinical trials evaluating B7-H4 based therapeutics become available, a comprehensive analysis of the combined safety, PK, and anti-tumour activity of PF-07260437 and other B7-H4 based entities currently in development will provide additional context on their relevance in cancer therapy. In recent years, there has been a growing interest in development of ADCs designed to specifically target and deliver cytotoxic drugs to cancer cells, thereby killing them [42]. In this regard, FIH clinical trial assessing the role of B7-H4 based ADCs in solid tumours, such as *XMT1660* (NCT05377996) appears promising based on the preclinical findings obtained from xenograft mice models [43]. This is reinforced by the preliminary clinical evidence from FIH clinical trials of *HS-20089* (NCT05263479) and *AZD8205* (NCT05123482) in patients with advanced solid tumours [44, 45]. Overall, publication of the findings of the above referenced clinical studies will advance our understanding of the efficacy and safety of the various B7-H4 based entities and eventually, reveal the true potential of B7-H4 based immunotherapeutic strategies. Finally, combining B7-H4 CD3 bispecific T-cell engager with other immunostimulatory agents, such as immunogenic ADCs, T cell therapies or cancer vaccination [46] may also increase the functional T cell density in solid tumours and overcome the immunosuppressive TMEs to unleash the complete cytotoxic potential of CD3 engagers as well as create further synergy for treatment of solid tumours.

## Conclusion

Overall, PF-07260437 demonstrated a manageable safety profile. CRS was successfully mitigated by dose priming strategy. However, the mechanism of action for transient liver enzyme elevation remained unclear. MTD was not identified. Dose escalation cohorts exhibited limited antitumour activity, with the best overall response being stable diseases in breast, ovarian, and endometrial cancers. Further preclinical and clinical research targeting B7-H4 through various mechanisms of action is needed to provide additional data and insights and improve benefit-to-risk ratio in the treatment of solid tumours.

## Supplementary Information

Below is the link to the electronic supplementary material.

Supplementary file1 (DOCX 74.1 KB)

## Data Availability

Upon request, and subject to review, Pfizer will provide the data that support the findings of this study. Subject to certain criteria, conditions, and exceptions, Pfizer may also provide access to the related individual de-identified participant data. See https://www.pfizer.com/science/clinical-trials/data-and-results for more information.
