# Supplementary material for: A phase 1 study of PF-07260437, a B7-H4 × CD3 bispecific T-cell engager, in patients with advanced or metastatic breast, ovarian, and endometrial cancer
Source: Invest New Drugs. 2026 May 22;44(4):305–16. doi: 10.1007/s10637-026-01614-2 (PMC13486042; doi:10.1007/s10637-026-01614-2)
Supplement: Supplementary file 1 [file 10637_2026_1614_MOESM1_ESM.docx]

**Supplementary Data**

# **Title**

**A phase 1 study of PF-07260437, a B7-H4 × CD3 bispecific T-cell engager, in patients with advanced or metastatic breast, ovarian, and endometrial cancer**

**Journal name:** *Investigational New Drugs*

# **Authors**

Fengting Yan^1*^, Marcia Cruz-Correa^2^, Jennifer Specht^3^, Edward Wenge Wang^4^, Jinyu Lu^5^, Hatem Soliman^6^, Amy Jackson-Fisher^7^, Szu-Yu Tang^7^, Sibo Jiang^7^, Christophe Le Corre^7^, Meng Li^8^, David Sommerhalder^9^

# **Affiliations**

^1^Swedish Cancer Institute, Seattle, WA 98104, United States; ^2^Pan American Center for Oncology Trials, 150 Av. Centro Médico, San Juan, 00935, Puerto Rico; ^3^Fred Hutchinson Cancer Center, 1100 Fairview Ave N, Seattle, WA 98109, United States; ^4^City of Hope Comprehensive Cancer Center, 1500 E Duarte Rd, Duarte, CA 91010, United States; ^5^Montefiore Einstein Comprehensive Cancer Center, 1695 Eastchester Rd, Bronx, NY 10461, United States; ^6^Moffitt Cancer Center, 12902 USF Magnolia Drive, Tampa, FL 33612, United States; ^7^Pfizer, Torrey Heights, 11202 El Camino Real, San Diego, CA 92130, United States; ^8^Pfizer, 181 Oyster Point Blvd, South San Francisco, CA 94080, United States; ^9^NEXT Oncology, 2829 Babcock Rd Suite 300, San Antonio, TX 78229, United States

*Corresponding author: Fengting Yan

**Mailing address:** Swedish Cancer Institute, 1221 Madison Street, Seattle, WA 98104, United States

**E-mail:** [fengting.yan@swedish.org](mailto:fengting.yan@swedish.org); [yfengtin@fredhutch.org](mailto:yfengtin@fredhutch.org)

# **Fig. S1** Patient disposition

# **Table S1** Summary of baseline demographic and clinical characteristics in patients who received at least one dose of PF-07260437 (safety analysis set)

| **Characteristic** | **Overall (n=30)** | **100 µg**  **(n=3)** | **300 µg (n=4)** | **500 µg**  **(n=4)** | **200 µg (priming) + 500 µg**  **(n=6)** | **200 µg (priming) + 800 µg**  **(n=4)** | **100 µg (1^st^ priming) + 300 µg (2^nd^ priming) + 800 µg**  **(n=4)** | **200 µg (priming) + 1600 µg**  **(n=5)** |  |
| --- | --- | --- | --- | --- | --- | --- | --- | --- | --- |
| ***Age, years, median (range)*** | 61.0  (41.0, 75.0) | 58.0  (44.0, 65.0) | 66.0  (60.0, 73.0) | 58.0  (52.0, 66.0) | 58.0 (41.0,75.0) | 67.0 (56.0,73.0) | 63.5  (58.0, 69.0) | 60.0  (47.0, 67.0) |  |
| ***Gender*** | | | | | | | | | |
| Female | 30 (100.0) | 3 (100.0) | 4 (100.0) | 4 (100.0) | 6 (100.0) | 4 (100.0) | 4 (100.0) | 5 (100.0) |  |
| ***Race*** | | | | | | | | | |
| White | 25 (83.3) | 3 (100.0) | 4 (100.0) | 4 (100.0) | 5 (83.3) | 2 (50.0) | 3 (75.0) | 4 (80.0) |  |
| Black/African American | 1 (3.3) | 0 (0) | 0 (0) | 0 (0) | 1 (16.7) | 0 (0) | 0 (0) | 0 (0) |  |
| Asian | 2 (6.7) | 0 (0) | 0 (0) | 0 (0) | 0 (0) | 1 (25.0) | 1 (25.0) | 0 (0) |  |
| Not reported | 2 (6.7) | 0 (0) | 0 (0) | 0 (0) | 0 (0) | 1 (25.0) | 0 (0) | 1 (20.0) |  |
| ***Ethnicity*** | | | | | | | | | |
| Hispanic or Latino | 9 (30.0) | 0 (0) | 1 (25.0) | 1 (25.0) | 3 (50.0) | 1 (25.0) | 2 (50.0) | 1 (20.0) |  |
| Others | 21 (70.0) | 3 (100.0) | 3 (75.0) | 3 (75.0) | 3 (50.0) | 3 (75.0) | 2 (50.0) | 4 (80.0) |  |
| ***ECOG PS*** | | | | | | | | | |
| 0 | 20 (66.7) | 3 (100.0) | 3 (75.0) | 2 (50.0) | 1 (16.7) | 3 (75.0) | 4 (100.0) | 4 (80.0) |  |
| 1 | 10 (33.3) | 0 (0) | 1 (25.0) | 2 (50.0) | 5 (83.3) | 1 (25.0) | 0 (0) | 1 (20.0) |  |
| ***Primary diagnosis*** | | | | | | | | | |
| Endometrial cancer | 5 (16.7) | 0 (0) | 1 (25.0) | 1 (25.0) | 1 (16.7) | 2 (50.0) | 0 (0) | 0 (0) |  |
| HER2+ breast cancer | 1 (3.3) | 0 (0) | 0 (0) | 1 (25.0) | 0 (0) | 0 (0) | 0 (0) | 0 (0) |  |
| HR+ HER2- breast cancer | 7 (23.3) | 1 (33.3) | 1 (25.0) | 2 (50.0) | 1 (16.7) | 0 (0) | 1 (25.0) | 1 (20.0) |  |
| Ovarian cancer | 14 (46.7) | 1 (33.3) | 2 (50.0) | 0 (0) | 4 (66.7) | 2 (50.0) | 3 (75.0) | 2 (40.0) |  |
| Triple negative breast cancer | 3 (10.0) | 1 (33.3) | 0 (0) | 0 (0) | 0 (0) | 0 (0) | 0 (0) | 2 (40.0) |  |
| ***#Prior systemic lines of therapy in setting, median (range)*** | | | | | | | | |  |
| Any setting | 4.0  (1.0, 23.0) | 10.0  (4.0, 12.0) | 3.0  (1.0, 7.0) | 7.0  (6.0, 11.0) | 4.5  (2.0, 23.0) | 2.5  (2.0, 3.0) | 3.5  (1.0, 6.0) | 5.0  (3.0, 9.0) |  |
| Advanced/metastatic setting | 3.5  (0, 11.0) | 6.0  (1.0, 8.0) | 2.0  (1.0, 6.0) | 6.5  (2.0, 11.0) | 4.0  (0.0, 11.0) | 1.5  (0.0, 2.0) | 2.5  (1.0, 4.0) | 4.0  (1.0, 7.0) |  |
| Chemotherapy in advanced/metastatic setting | 2.0  (0.0, 6.0) | 4.0  (1.0, 4.0) | 1.5  (0.0, 3.0) | 2.5  (1.0, 6.0) | 2.0  (0.0, 6.0) | 1.5  (0.0, 2.0) | 1.5  (0.0, 2.0) | 2.0  (0.0, 5.0) |  |
| Immunotherapy in advanced/metastatic setting | 1.0  (0.0, 7.0) | 3.0  (0.0, 3.0) | 0.5  (0.0, 1.0) | 0.5  (0.0, 5.0) | 2  (0.0, 7.0) | 0.5  (0.0, 1.0) | 0.5  (0.0, 2.0) | 1.0  (1.0, 2.0) |  |

All values are presented as n (%), unless stated otherwise. Chemotherapy includes medications coded under ATC Level 3 Codes: L01A, L01B, L01C, L01D and ATC Level 4 codes: L01XA, L01XX, L01XY, and excludes BMS 986301. Immunotherapy includes medications coded under ATC Level 3 code: L01F and ATC Level 4 code: L01XX with BMS 986301.

ATC, Anatomical Therapeutic Chemical; ECOG PS, Eastern Cooperative Oncology Group Performance Status; HER2-, human epidermal growth factor receptor 2 negative; HR, hormone receptor.

**Table S2** DLTs (Grade 1-2 and Grade ≥ 3) by PT and maximum CTCAE Grade (All causalities, DLT observation period) (safety analysis set)

| **Number of patients evaluable for AEs** | **100 µg SC Q2W**  **(N=3)** | | **300 µg SC Q2W**  **(N=4)** | | **500 µg SC Q2W**  **(N=4)** | | **200 µg (priming) +**  **500 µg SC Q2W (N=6)** | |
| --- | --- | --- | --- | --- | --- | --- | --- | --- |
|  | **Grade 1-2** | **Grade ≥3** | **Grade 1-2** | **Grade ≥3** | **Grade 1-2** | **Grade ≥3** | **Grade 1-2** | **Grade ≥3** |
| Number (%) of patients by PT | n (%) | n (%) | n (%) | n (%) | n (%) | n (%) | n (%) | n (%) |
| Patients with events | 0 | 0 | 0 | 0 | 0 | 1 (25.0) | 0 | 0 |
| ALT increased | 0 | 0 | 0 | 0 | 0 | 0 | 0 | 0 |
| AST increased | 0 | 0 | 0 | 0 | 0 | 0 | 0 | 0 |
| Blood bilirubin increased | 0 | 0 | 0 | 0 | 0 | 0 | 0 | 0 |
| CRS | 0 | 0 | 0 | 0 | 0 | 1 (25.0) | 0 | 0 |
| Total PT events | 0 | 0 | 0 | 0 | 0 | 1 | 0 | 0 |
| **Number of patients evaluable for AEs** | **200 µg (priming) +**  **800 µg SC Q2W**  **(N=4)** | | **100 µg (priming) +**  **300 µg (second priming) + 800 µg SC Q2W (N=4)** | | **200 µg (priming) +**  **1600 µg SC Q2W**  **(N=5)** | | **Total**  **(N=30)** | |
|  | **Grade 1-2** | **Grade ≥3** | **Grade 1-2** | **Grade ≥3** | **Grade 1-2** | **Grade ≥3** | **Grade 1-2** | **Grade ≥3** |
| Number (%) of patients by PT | n (%) | n (%) | n (%) | n (%) | n (%) | n (%) | n (%) | n (%) |
| Patients with events | 0 | 0 | 0 | 2 (50.0) | 0 | 1 (20.0) | 0 | 4 (13.3) |
| ALT increased | 0 | 0 | 0 | 1 (25.0)^a^ | 0 | 1 (20.0) | 0 | 2 (6.7) |
| AST increased | 0 | 0 | 1 (25.0) | 1 (25.0)^b^ | 0 | 0 | 1 (3.3) | 1 (3.3) |
| Blood bilirubin increased | 0 | 0 | 1 (25.0)^c^ | 0 | 0 | 0 | 1 (3.3) | 0 |
| CRS | 0 | 0 | 0 | 0 | 0 | 0 | 0 | 1 (3.3) |
| Total PT events | 0 | 0 | 2 | 2 | 0 | 1 | 2 | 4 |

^a^2 episodes of ALT increased and worsened from Grade 2 to Grade 3.

^b^Grade 2 AST increased eventually became Grade 3 and qualified as a DLT.

^c^Prolonged dose interruptions due to increase in Grade 2 blood bilirubin.

Patients reporting more than 1 DLT within a PT are counted only once in that PT at the maximum grade. DLTs are sorted in descending frequency by the total column of each treatment group. Includes data up to earliest of 30 days post last dosing date and 1 day prior to new anti-cancer therapy. MedDRA v26.1 coding dictionary applied, and severity graded by NCI CTCAE version 5.

AE, adverse event; ALT, alanine aminotransferase; AST, aspartate aminotransferase; CRS, cytokine release syndrome; CTCAE, Common Terminology Criteria for Adverse Events; DLT, dose-limiting toxicity; MedDRA, Medical Dictionary for Regulatory Activities; NCI, National Cancer Institute; PT, Preferred Term; Q2W, every 2 weeks; SC, subcutaneous.

# **Table S3** Treatment-emergent adverse events (in >5% patients) by preferred term and maximum CTCAE Grade in patients who received at least one dose of PF-07260437 (safety analysis set)

| **Number (%) of patients:**  **by preferred term** | **Grade 1** | **Grade 2** | **Grade 3** | **Grade 4** | **Grade 5** | **Total** |
| --- | --- | --- | --- | --- | --- | --- |
| Patients with events | 3 (10.0) | 5 (16.7) | 19 (63.3) | 1 (3.3) | 2 (6.7) | 30 (100.0) |
| Aspartate aminotransferase increased | 5 (16.7) | 7 (23.3) | 9 (30.0) | 0 | 0 | 21 (70.0) |
| Alanine aminotransferase increased | 1 (3.3) | 5 (16.7) | 14 (46.7) | 0 | 0 | 20 (66.7) |
| Blood alkaline phosphatase increased | 4 (13.3) | 7 (23.3) | 7 (23.3) | 0 | 0 | 18 (60.0) |
| Fatigue | 10 (33.3) | 5 (16.7) | 0 | 0 | 0 | 15 (50.0) |
| Blood bilirubin increased | 2 (6.7) | 11 (36.7) | 1 (3.3) | 0 | 0 | 14 (46.7) |
| Injection site reaction | 13 (43.3) | 0 | 0 | 0 | 0 | 13 (43.3) |
| Anaemia | 6 (20.0) | 3 (10.0) | 2 (6.7) | 0 | 0 | 11 (36.7) |
| Pruritus | 7 (23.3) | 1 (3.3) | 0 | 0 | 0 | 8 (26.7) |
| Vomiting | 4 (13.3) | 3 (10.0) | 1 (3.3) | 0 | 0 | 8 (26.7) |
| Constipation | 5 (16.7) | 2 (6.7) | 0 | 0 | 0 | 7 (23.3) |
| Cytokine release syndrome | 5 (16.7) | 1 (3.3) | 1 (3.3) | 0 | 0 | 7 (23.3) |
| Decreased appetite | 5 (16.7) | 2 (6.7) | 0 | 0 | 0 | 7 (23.3) |
| Dyspnoea | 6 (20.0) | 0 | 0 | 0 | 1 (3.3) | 7 (23.3) |
| Headache | 7 (23.3) | 0 | 0 | 0 | 0 | 7 (23.3) |
| Hypomagnesaemia | 7 (23.3) | 0 | 0 | 0 | 0 | 7 (23.3) |
| Hyponatraemia | 3 (10.0) | 4 (13.3) | 0 | 0 | 0 | 7 (23.3) |
| Nausea | 2 (6.7) | 4 (13.3) | 0 | 0 | 0 | 6 (20.0) |
| Back pain | 2 (6.7) | 3 (10.0) | 0 | 0 | 0 | 5 (16.7) |
| Cough | 4 (13.3) | 1 (3.3) | 0 | 0 | 0 | 5 (16.7) |
| Pyrexia | 5 (16.7) | 0 | 0 | 0 | 0 | 5 (16.7) |
| Dehydration | 1 (3.3) | 3 (10.0) | 0 | 0 | 0 | 4 (13.3) |
| Hypokalaemia | 2 (6.7) | 1 (3.3) | 0 | 1 (3.3) | 0 | 4 (13.3) |
| Lipase increased | 3 (10.0) | 1 (3.3) | 0 | 0 | 0 | 4 (13.3) |
| Lymphocyte count decreased | 0 | 1 (3.3) | 3 (10.0) | 0 | 0 | 4 (13.3) |
| Rash maculo-papular | 4 (13.3) | 0 | 0 | 0 | 0 | 4 (13.3) |
| Abdominal distension | 2 (6.7) | 1 (3.3) | 0 | 0 | 0 | 3 (10.0) |
| Abdominal pain | 1 (3.3) | 2 (6.7) | 0 | 0 | 0 | 3 (10.0) |
| Abdominal pain upper | 2 (6.7) | 1 (3.3) | 0 | 0 | 0 | 3 (10.0) |
| Ascites | 0 | 3 (10.0) | 0 | 0 | 0 | 3 (10.0) |
| Diarrhoea | 2 (6.7) | 1 (3.3) | 0 | 0 | 0 | 3 (10.0) |
| Hyperglycaemia | 3 (10.0) | 0 | 0 | 0 | 0 | 3 (10.0) |
| Hypoalbuminemia | 2 (6.7) | 1 (3.3) | 0 | 0 | 0 | 3 (10.0) |
| Nasal congestion | 3 (10.0) | 0 | 0 | 0 | 0 | 3 (10.0) |
| Non-cardiac chest pain | 2 (6.7) | 1 (3.3) | 0 | 0 | 0 | 3 (10.0) |
| Pleural effusion | 1 (3.3) | 2 (6.7) | 0 | 0 | 0 | 3 (10.0) |
| Urinary tract infection | 0 | 3 (10.0) | 0 | 0 | 0 | 3 (10.0) |
| White blood cell count decreased | 0 | 3 (10.0) | 0 | 0 | 0 | 3 (10.0) |
| Arthralgia | 2 (6.7) | 0 | 0 | 0 | 0 | 2 (6.7) |
| Blood lactate dehydrogenase increased | 2 (6.7) | 0 | 0 | 0 | 0 | 2 (6.7) |
| Chills | 2 (6.7) | 0 | 0 | 0 | 0 | 2 (6.7) |
| Fall | 2 (6.7) | 0 | 0 | 0 | 0 | 2 (6.7) |
| Flank pain | 0 | 2 (6.7) | 0 | 0 | 0 | 2 (6.7) |
| Hypercalcaemia | 1 (3.3) | 0 | 1 (3.3) | 0 | 0 | 2 (6.7) |
| Hypertension | 1 (3.3) | 1 (3.3) | 0 | 0 | 0 | 2 (6.7) |
| Hypertransaminasemia | 0 | 0 | 2 (6.7) | 0 | 0 | 2 (6.7) |
| Influenza like illness | 2 (6.7) | 0 | 0 | 0 | 0 | 2 (6.7) |
| Muscle spasms | 2 (6.7) | 0 | 0 | 0 | 0 | 2 (6.7) |
| Neutrophil count decreased | 2 (6.7) | 0 | 0 | 0 | 0 | 2 (6.7) |
| Oropharyngeal pain | 2 (6.7) | 0 | 0 | 0 | 0 | 2 (6.7) |
| Pain | 2 (6.7) | 0 | 0 | 0 | 0 | 2 (6.7) |
| Upper respiratory tract infection | 0 | 2 (6.7) | 0 | 0 | 0 | 2 (6.7) |
| Vertigo | 1 (3.3) | 0 | 1 (3.3) | 0 | 0 | 2 (6.7) |

TEAEs are all causality unless otherwise specified. Patients reporting >1 TEAE within a PT are counted only once in that PT at the maximum grade. TEAEs are sorted in descending frequency by the total column of Total treatment group. Includes data up to earliest of 30 days post last dosing date and 1 day prior to new anti-cancer therapy. MedDRA v26.1 coding dictionary applied, and severity graded by NCI CTCAE version 5.

CTCAE, Common Terminology Criteria for Adverse Events; MedDRA, Medical Dictionary for Regulatory Activities; NCI, National Cancer Institute; PT, preferred term; TEAE, Treatment-emergent adverse event.

# **Table S4** Treatment-related TEAEs (in >5% patients) by preferred term and Maximum CTCAE Grade in patients who received at least one dose of PF-07260437 (safety analysis set)

| **Number (%) of patients:**  **by preferred term** | **Grade 1** | **Grade 2** | **Grade 3** | **Total** |
| --- | --- | --- | --- | --- |
| Patients with events | 4 (13.3) | 4 (13.3) | 21 (70.0) | 29 (96.7) |
| Aspartate aminotransferase increased | 5 (16.7) | 7 (23.3) | 9 (30.0) | 21 (70.0) |
| Alanine aminotransferase increased | 1 (3.3) | 5 (16.7) | 14 (46.7) | 20 (66.7) |
| Blood alkaline phosphatase increased | 4 (13.3) | 7 (23.3) | 7 (23.3) | 18 (60.0) |
| Blood bilirubin increased | 2 (6.7) | 10 (33.3) | 1 (3.3) | 13 (43.3) |
| Injection site reaction | 13 (43.3) | 0 | 0 | 13 (43.3) |
| Fatigue | 6 (20.0) | 4 (13.3) | 0 | 10 (33.3) |
| Cytokine release syndrome | 5 (16.7) | 1 (3.3) | 1 (3.3) | 7 (23.3) |
| Pruritus | 6 (20.0) | 1 (3.3) | 0 | 7 (23.3) |
| Anaemia | 4 (13.3) | 0 | 1 (3.3) | 5 (16.7) |
| Headache | 5 (16.7) | 0 | 0 | 5 (16.7) |
| Pyrexia | 5 (16.7) | 0 | 0 | 5 (16.7) |
| Decreased appetite | 4 (13.3) | 0 | 0 | 4 (13.3) |
| Lymphocyte count decreased | 0 | 1 (3.3) | 3 (10.0) | 4 (13.3) |
| Nausea | 2 (6.7) | 2 (6.7) | 0 | 4 (13.3) |
| Rash maculo-papular | 4 (13.3) | 0 | 0 | 4 (13.3) |
| Vomiting | 1 (3.3) | 2 (6.7) | 1 (3.3) | 4 (13.3) |
| Lipase increased | 2 (6.7) | 1 (3.3) | 0 | 3 (10.0) |
| Nasal congestion | 3 (10.0) | 0 | 0 | 3 (10.0) |
| Abdominal pain | 1 (3.3) | 1 (3.3) | 0 | 2 (6.7) |
| Chills | 2 (6.7) | 0 | 0 | 2 (6.7) |
| Diarrhoea | 1 (3.3) | 1 (3.3) | 0 | 2 (6.7) |
| Hyperglycaemia | 2 (6.7) | 0 | 0 | 2 (6.7) |
| Hypertransaminasemia | 0 | 0 | 2 (6.7) | 2 (6.7) |
| Hyponatraemia | 0 | 2 (6.7) | 0 | 2 (6.7) |
| Influenza like illness | 2 (6.7) | 0 | 0 | 2 (6.7) |
| Oropharyngeal pain | 2 (6.7) | 0 | 0 | 2 (6.7) |
| White blood cell count decreased | 0 | 2 (6.7) | 0 | 2 (6.7) |

Grade 4 or 5 treatment related TEAEs were not reported in this study. Treatment related means related to any study intervention. Patients reporting >1 treatment-related TEAE within a PT are counted only once in that PT at the maximum grade. Treatment-related TEAEs are sorted in descending frequency by the total column of Total treatment group. Includes data up to earliest of 30 days post last dosing date and 1 day prior to new anti-cancer therapy. MedDRA v26.1 coding dictionary applied, and severity graded by NCI CTCAE version 5.

CTCAE, Common Terminology Criteria for Adverse Events; MedDRA, Medical Dictionary for Regulatory Activities; NCI, National Cancer Institute; PT, preferred term; TEAE, Treatment-emergent adverse event.
